# Bioaerosol assessment of indoor air in hospital wards for isolation of *Nocardia* species from a tertiary care hospital in Iranshahr, Iran

**DOI:** 10.1016/j.nmni.2025.101689

**Published:** 2025-12-15

**Authors:** Zahed Ahmadi, Alireza Moradabadi, Sara Kamal-Shasavar

**Affiliations:** a Tropical and Communicable Diseases Research Center, Iranshahr University of Medical Sciences, Iranshahr, Iran; b Department of Laboratory Medicine, Khomein University of Medical Science, Khomein, Iran; c Student of Research Committee, Department of Hematology, Faculty of Medical Sciences, Tarbiat Modares University, Tehran, Iran; d Molecular and Medicine Research Center, Khomein University of Medical Sciences, Khomein, Iran; e Department of Microbiology, School of Medicine, Mashhad University of Medical Sciences, Mashhad, Iran

**Keywords:** Airborne nanoparticles, Hospital indoor air, *Nocardia species*

## Abstract

**Background:**

Bioaerosols can be a critical role in the transmission of hospital-acquired infections. *Nocardia species* are opportunistic pathogens that primarily affect immunocompromised patients, accounting for approximately 1–2% of all hospital-acquired bacterial infections in this population. To date, there are no comprehensive studies examining the presence of *Nocardia* in hospital indoor air. This study aimed to assess the species diversity of the *Nocardia* genus in different hospital indoor environments at Khatam Hospital, Iranshahr, Iran.

**Methods:**

Particle concentration in various hospital wards was measured using the direct reading method. Bioaerosol sampling followed NIOSH methods 0800 and 0801, using Sauton's medium plates. Each Petri dish was incubated in an inverted position for three weeks at both 25 °C and 37 °C in parallel. *Nocardia* isolates were identified through phenotypic tests, including growth in lysozyme broth and substrate degradation assays (tyrosine, xanthine, and hypoxanthine), followed by molecular confirmation.

**Results:**

The orthopedic ward exhibited the highest particle concentration among all wards. Fourteen *Nocardia* isolates were recovered: four from the emergency department, four from infectious diseases, and three from surgery, two from hemodialysis, and one from orthopedics. The identified species included *N. cyriacigeorgica*, *N. asteroides*, *N. otitidiscaviarum*, *N. wallacei*, *N. kroppenstedtii*, *N. farcinica*, and *N. nova*.

**Conclusion:**

This study represents one of the earliest documented investigations, detecting clinically relevant *Nocardia species* in hospital indoor air. Although the direct link between airborne *Nocardia* and hospital-acquired infections remains to be proven, the detection of pathogenic species in patient-care environments underscores a potential risk to vulnerable individuals.

## 1 Introduction

Bioaerosols are defined as airborne particles of biological origin that may occur in solid, liquid, or gaseous phases. They are derived from microorganisms, plants, or animals and can include a wide range of viable or non-viable entities such as bacteria, bacterial endotoxins, fungi, viruses, high-molecular-weight allergens, pollen grains, and fragments of plant or animal matter suspended in the atmosphere [1]. Various human activities, including talking, sneezing, coughing, walking, and other daily actions, can generate and release airborne bacteria. The number of occupants in an indoor environment also significantly influences the concentration of these bioaerosol particles. Because bioaerosols originate from multiple and diverse sources, characterizing their composition and assessing their microbial contamination remain challenging actions [1]. Bioaerosols are associated with a wide range of health effects, including hospital-acquired infections, which can threaten the health of hospitalized patients and their companions [2]. Immunocompromised individuals are generally more susceptible to infections compared to the general population. Pathogenic bioaerosols, such as bacteria, constitute a fraction of airborne particles classified as PM10 [3]. This issue raises concerns about the significant role air may play in disease transmission [4]. Hospital environments represent critical settings for the transmission of infectious agents. Due to the presence of patients and potential pathogens, the assessment of bioaerosol concentration plays a vital role in evaluating indoor air quality and in the prevention and control of hospital-acquired infections [5]. Currently, airborne particle levels are reported in terms of weight or particle count per unit volume of air [6]. A more detailed analysis of aerosols involves studying the particle size distribution based on their number [6,7]. In addition to quantifying bioaerosol concentrations, identifying the pathogenic microbial communities suspended in hospital air is essential for infection control [8].

Edmond Nocard initially defined the genus *Nocardia*, which belongs to the family *Nocardiaceae*, in 1888 [9]. These bacteria are Gram-positive, filamentous, aerobic, somewhat acid-fast, opportunistic, and can be found in a variety of environments. Human nocardiosis is mainly caused by inhaling *Nocardia* aerosols or direct inoculation through injured skin [10]. The most frequent form is pulmonary nocardiosis, which primarily affects immunocompromised individuals such as those with HIV/AIDS, cancer, diabetes, or organ transplants [11]. Although *Nocardia* infections are mainly opportunistic, they can cause severe pulmonary, cutaneous, central nervous system, and widespread infections [12]. *Nocardia* is not found in the typical human or animal flora; hence, human-to-human transmission has not been documented [10,13]. However, infection with this pathogen is widespread in both people and animals [14]. According to the literature, 1000 *Nocardia* infections are documented each year in the United States [15]. Infection is mostly connected with environmental exposure to polluted soil, air, dust, and water [16,17].

Several studies worldwide have attempted to detect *Nocardia species* in air or dust samples from hospital and non-hospital environments using diverse methodologies. Culture-based studies have successfully isolated *Nocardia* from soil and dust but are limited by the slow growth rate and overgrowth of faster-growing microbes [14]. Molecular methods, such as polymerase chain reaction (PCR) assays targeting 16S rRNA or *hsp 65* genes, have improved detection sensitivity and species-level identification [10,18]. More recently, metagenomic sequencing has been used to characterize airborne microbial communities, including *Nocardia*, providing a comprehensive overview of microbial diversity in healthcare settings [19]. However, most of these studies have been conducted in temperate, high-resource countries and are often limited by small sample sizes, short monitoring durations, or restricted environmental coverage. Consequently, data regarding *Nocardia*-containing bioaerosols in hospitals, particularly in developing or tropical regions, remain extremely scarce.

Given the growing population of immunocompromised individuals, surveillance of hospital air for opportunistic pathogens is increasingly important [20,21]. Periodic microbiological surveillance of hospital indoor air can increase awareness of environmental reservoirs of opportunistic pathogens and guide targeted infection-control measures in high-risk wards [22]. Another study identified bioaerosols as the most significant source of hospital-acquired infections. These bioaerosols can contribute to prolonged hospital stays, the emergence of drug-resistant microorganisms, increased treatment costs, and higher mortality rates. Recent data suggest that healthcare-associated infections (HAIs) affect approximately 3.2 % of hospitalized patients in the United States—equivalent to about 687,000 cases (1 in 31 patients)—and around 6.5 % of patients in the European Union/European Economic Area. The global prevalence is likely to surpass these figures [5,23].

Recent metagenomic and amplicon-based studies demonstrate that hospital air and dust harbor complex microbial communities and inhalable antibiotic-resistance gene (ARG) reservoirs, underscoring the need for targeted airborne surveillance to detect clinically relevant organisms such as *Nocardia* [24,25]. Despite reports of *Nocardia* isolation from hospital equipment and environmental sources globally, there is a marked lack of data from healthcare facilities in developing countries such as Iran. Because infections caused by *Nocardia* are often underdiagnosed due to slow identification methods and limited laboratory capacity [26]. Iranshahr, situated in southeastern Iran, is characterized by a hot and arid climate. The city is located at an elevation of approximately 591 m above sea level, with an average annual temperature of 26.8 °C and a mean relative humidity of 30 %. Due to the region's socioeconomic deprivation and the occurrence of the seasonal 120-day monsoon winds—commonly accompanied by dust storms—environmental conditions may facilitate the emergence and transmission of *Nocardia* infections [27]. This research gap poses potential risks to patient safety. Therefore, this study aimed to monitor aerosol levels in terms of particle weight and count per unit air volume and to investigate the species diversity of *Nocardia* in aerosols collected from various hospital wards in Khatam Hospital, Iranshahr, Iran.

## 2 Materials and methods

### 2.1 Study setting

This descriptive-analytical study was conducted between March 2023 and June 2024 across 12 wards of Iranshahr Khatam Hospital. Khatam Hospital is a 220-bed tertiary-care public facility and serves as the main referral center for Iranshahr and its surrounding areas, with a catchment population of approximately 250,000. The hospital provides a wide range of services, including general medicine, surgery, orthopedics, hemodialysis, infectious diseases, and emergency care. An estimated 15–20 % of admitted patients are immunocompromised, due to conditions such as solid organ or hematologic malignancies, HIV infection, complicated diabetes, or long-term corticosteroid therapy. Each ward sampled contains 15–30 beds, with inter-bed spacing of 1–2 m, and is ventilated using split air conditioners. None of the wards are equipped with HEPA filtration systems or controlled negative/positive pressure environments. During the study period, powdered latex gloves were still in use in some wards. In addition, daily plaster cast application and removal procedures were performed in the orthopedic ward.

### 2.2 Sample collection process

During the study we checked about construction around the hospital for potential release of dust, so there was no construction on-site and around 200 m around the hospital. A total of 240 dust (particulate matter) assessments were performed during three sampling sessions at the beginning, middle, and end of each month using a calibrated Met One GT-526S particle counter (USA). For bacterial contamination monitoring, particularly for the hard-to-grow *Nocardia species*, 108 biological air samples were collected following NIOSH methods 0800 and 0801. Air samples were drawn for 10 min at a flow rate of 28.3 L/min over Sauton's medium enriched with kanamycin, nystatin, and nalidixic acid (50 μL of each per mL of medium). To prevent bacterial cross-contamination, the two-stage Andersen biosampler was disinfected with 70 % ethanol before each sampling. After labeling, the samples were incubated in Sauton's media (containing kanamycin, nystatin, and nalidixic acid) for three weeks at 25 °C and 37 °C in an atmosphere of 5 % CO_2_.

### 2.3 Microbiological evaluations

Colonies grown on culture media were identified as *Nocardia* based on colony morphology, Kinyoun staining, growth rate, pigment production, and biochemical tests, including growth in lysozyme broth and the hydrolysis of tyrosine, xanthine, and hypoxanthine [10].

### 2.4 Molecular identification

Chromosomal DNA was extracted using a simple boiling method [28]. Briefly, colonies were suspended in 200 μL of TE buffer, boiled for 30 min, and centrifuged at 11,800×*g* for 10 min. The supernatant was transferred to a sterile microtube and centrifuged again at 20,000×*g* for 10 min. The extracted DNA was stored in 50 μL of Milli-Q water for further molecular assessment. The 16S rRNA gene was amplified using universal primers 27-F (5′-AGAGTTTGATCCTGGCTCAG-3′) and 1492-R (5′-GGTTACCTTGTTACGACTT-3′). Polymerase Chain Reaction (PCR) was performed in a final volume of 25 μL containing 2 μL of 10X buffer, 0.8 μL of MgCl_2_, 0.4 μL of dNTP mix, 0.5 μL of each primer (10 pmol), 0.25 μL of Taq DNA Polymerase, 2.5 μL of template DNA, and sterile distilled water. The thermal cycling conditions were as follows: initial denaturation at 95 °C for 4 min, followed by 30 cycles (denaturation at 95 °C for 1 min, annealing at 60 °C for 30 s, and extension at 72 °C for 35 s), as well as final extension at 72 °C for 5 min [29]. The PCR product was sent to Bioneer Company (South Korea) for direct sequencing using an ABI 3100 genetic analyzer. The target sequence was aligned with the relevant sequences of *Nocardia* retrieved from the GenBank database using the online nucleotide BLAST server. In the next step, a phylogenetic tree was obtained by use of the NJ method K2P distance correction model corresponding to 1000 bootstrap replications; it was drawn through MEGA 4.1 software.

## 3 Results

### 3.1 Monitoring of particulate matter (PM)

Airborne particles are categorized based on their aerodynamic diameter, which determines their ability to penetrate the respiratory tract. PM 10 refers to coarse particles with diameters of 10 μm (μm) or less. Fine particles (PM 2.5) are defined as those with diameters of 2.5 μm or less, while ultrafine particles (PM0.1) measure 100 nm (nm) or less. Fine particles can penetrate deeply into the lungs, reaching the alveolar regions. Ultrafine particles, in turn, may partially penetrate the body through the alveolar-capillary membrane. Meanwhile *Nocardia* filaments and fragments are typically 1–10 μm in length, the size fraction 1–10 μm is most relevant for potential inhalation and pulmonary nocardiosis [10]. Table 1 shows the concentration of airborne particles with aerodynamic sizes more than 0.3 μm (PM0.3), 0.5 μm (PM0.5), 1 μm (PM1), 5 μm (PM5), and 10 μm (PM10) in the air of five hospital wards: infectious diseases, orthopedics, hemodialysis, emergency, and operating room 2. The number of suspended particles within particular size ranges can be computed by deducting the values between neighboring particle size categories. For instance, the orthopedics ward had the highest quantity of suspended particles per cubic meter of air (37,280,466 particles) with aerodynamic sizes between 0.3 and 0.5 μm.

**Table 1:** Concentration of particles in various hospital wards (number per cubic meter).

Ward	Environmental factor		Particle					
	RH%	T (^0^C)	PM 0.3 (N/m^3^)	PM 0.5 (N/m^3^)	PM 1 (N/m^3^)	PM 5 (N/m^3^)	PM 10 (N/m^3^)	PM1-PM10 (N/m^3^)
Orthopedics	36.1	32	58,532,202	21,251,736	5,954,080	188,743	27,905	5,926,175
Hemodialysis	50	29.3	54,544,799	11,324,502	3,992,582	141,999	19,663	3,972,919
Adults' emergency	48	29.7	36,921,817	9,885,788	4,192,629	271,164	58,165	4,134,464
Surgery Room 2	54	25	37,218,649	6,836,453	2,116,684	25,315	1295	2,115,389
Infectious Ward	46.2	29.7	33,492,875	6,772,636	2,395,031	76,651	15,896	2,379,135

PM: Particulate Matter RH: relative humidity T: temperature.

Additionally, the particle mass concentrations (by weight) for the five wards were analyzed and are summarized in Table 2. This includes the mass of particles smaller than 1 μm (PM1), 2.5 μm (PM2.5), 4 μm (PM4), 7 μm (PM7), 10 μm (PM10), and total suspended particles (TSP). It is important to note that as particle size decreases, more particles contribute to a single unit of weight, emphasizing the greater health significance of particle concentration in terms of count per unit volume of air.

**Table 2:** Concentration of particles in various hospital wards (micrograms per cubic meter).

Ward	Environmental factor		Particle					
	RH%	T (^0^C)	PM 1 (μg/m3)	PM 2.5 (μg/m3)	PM 4 (μg/m3)	PM 7 (μg/m3)	PM 10 (μg/m3)	TSP (μg/m3)
Orthopedics	36.1	32	17.3	55.30	111	180.73	221.23	262.50
Hemodialysis	50	29.3	8.9	33.97	78.47	125.70	150.77	178.70
Adults' emergency	48	29.7	7.3	29.33	74.80	152.40	205.77	288.03
Surgery Room	54	25	7.1	21.03	38.27	50.37	55.57	58.20
Infectious Ward	46.2	29.7	6.7	22.53	47.57	76.30	90.50	116.03

PM: Particulate Matter RH: relative humidity T: temperature.

### 3.2 Isolation and identification of nocardia

A total of 14 *Nocardia* isolates were obtained from the culture of 108 hospital dust samples. These isolates were recovered from the hemodialysis, emergency, orthopedics, operating room, and infectious disease wards. The ambient temperature during sample collection ranged from 25 to 32 °C, with relative humidity (RH) levels between 36.1 % and 54 %.

**Fig. 1:**
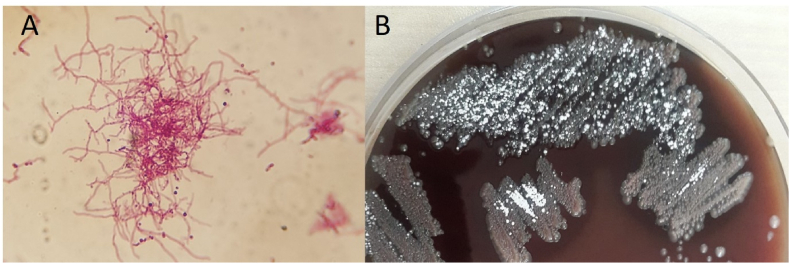
Kinyoun staining (A) and Colony (B) of *Nocardia* through phenotypic evaluations.

All 14 isolates exhibited partial acid-fast properties and resistance to lysozyme (Fig. 1). Molecular analysis of their 16S rRNA gene sequences revealed signature sequences characteristic of *Nocardia*, such as 70–98 (U-A), 139–224 (G-C), 843 (C), 1008–1021 (C-G), 1189 (C), 1244–129 (C-G), and 1308–1329 (C-G) [30].

Table 3 presents conventional phenotypic characteristics that, together with 16S rRNA sequencing, allowed definitive species identification. These tests (lysozyme resistance and arylsulfatase/substrate degradation patterns) remain widely used in resource-limited settings where molecular methods are unavailable. The primary characteristics of the *Nocardia* isolates are summarized in Table 3. The most frequently identified species was *N. cyriacigeorgica* (35.7 %), followed by *N. asteroides* (28.5 %). Single isolates (7.1 %) of *N. otitidiscaviarum*, *N. wallacei*, *N. kroppenstedtii*, *N. farcinica*, and *N. nova* were identified. Of these, four isolates were from the emergency ward, four from infectious diseases, three from surgery, two from hemodialysis, and one from the orthopedics ward.

**Table 3:** The conventional and molecular profile of *Nocardia* isolates from dust samples.

Isolate	Hospital ward	Temperature (^0^C)	pH	Lysozyme resistance	Hydrolysis			Similarity (%)	Species
					tyrosine	xanthine	hypoxanthine		
AK38	Emergency	26.2	7.1	+	–	+	+	99.8	*N. otitidiscaviarum*
AK56	Infectious diseases	27.6	7.6	+	–	–	–	99.9	*N. asteroides*
AK62	surgery	29.1	7.1	+	–	–	–	99.7	*N. asteroides*
AK73	Orthopedics	29.3	7.3	+	–	–	–	100	*N. cyriacigeorgica*
AK85	Emergency	27.6	7.6	+				9.8	*N. wallacei*
AK87	Emergency	30.3	7.3	+	–	–	–	100	*N. asteroides*
AK93	surgery	27.5	7.5	+	–	–	–	99.5	*N. cyriacigeorgica*
AK107	Infectious diseases	31.1	7.1	+	–	–	–	99.9	*N. cyriacigeorgica*
AK112	surgery	31.2	7.2	+	–	–	–	99.8	*N. kroppenstedtii*
AK115	Infectious diseases	30.5	7.5	+				99.5	*N. farcinica*
AK134	Emergency	29.3	7.3	+	–	–	–	100	*N. cyriacigeorgica*
AK142	Infectious diseases	25.5	7.5	+	–	–	–	99.9	*N. nova*
AK168	Hemodialysis	27.1	7.1	+	–	–	–	99.8	*N. asteroids*
AK171	Hemodialysis	28.3	7.6	+	–	–	–	99.8	*N. cyriacigeorgica*

### 3.3 Conventional and molecular profile of nocardia isolates

**Fig. 2:**
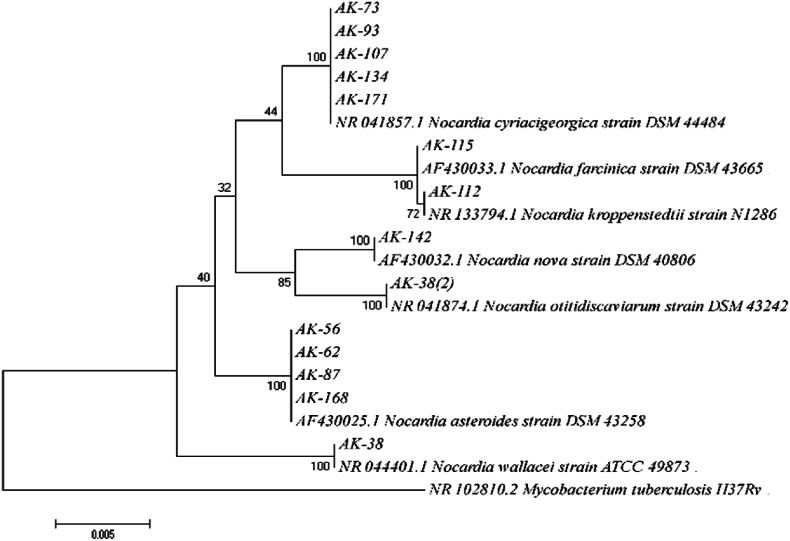
16S rRNA sequence-based phylogenetic tree of the *Nocardia* isolates from hospital dust samples and closely related species. Bootstrap percentages are indicated at each node. The tree was rooted with *Mycobacterium tuberculosis* H37Rv.

According to the molecular study, isolates AK73, AK93, AK107, AK134, and AK171 were 99.5–100 % similar to *N. cyriacigeorgica* DSM 44484. *N. asteroides* DSM 43258 and isolates AK56, AK62, AK87, and AK168 shared 99.7–100 % similarity. Isolate AK38 showed 99.8 % similarity to DSM 43242 for *N. otitidiscaviarum*. Isolate AK85 and *N. wallacei* ATCC 49873 showed 99.8 % similarity. The similarity between isolate AK112 and *N. kroppenstedtii* strain N1286 was 99.8 %. *N. farcinica* DSM 43665 showed 99.5 % similarity with isolate AK115. Isolate AK142 and *N. nova* DSM 40806 were 99.9 % identical. A phylogenetic tree built using 16S rRNA sequences and examined using the Neighbor-Joining (NJ) approach with the Kimura 2-Parameter (K2P) model verified the connection and proximity between these *Nocardia* isolates and their closest species. Each branch was verified by Bootstrap support (1000 replicates) (Fig. 2).

Considering that the filament length in various *Nocardia* strains ranges from 1 to 10 μm, the total number of particles within this range is presented in Table 1. The data in Table 1 indicate that there is no significant correlation between the number of particles ranging from 1 to 10 μm and the number of isolated *Nocardia species*. Spearman correlation analysis showed no statistically significant association between the number of particles in the 1–10 μm range and the number of *Nocardia* isolates recovered from the same ward (rs = 0.17, P = 0.78), confirming that total particle count is a poor surrogate for *Nocardia* bioaerosol concentration in this setting.

## 4 Discussion

Microbial monitoring of indoor air is a critical measure for assessing microbial contamination in hospital environments [31]. Determining the microbial concentration of airborne bacteria is essential for evaluating indoor air quality, particularly due to the health risks posed to immunocompromised individuals [32]. Unfortunately, there are no established global guidelines for acceptable microbial loads in hospital indoor air [33]. The permissible particle count in cleanroom environments is defined by the ISO 14644-1 standard. For particles with an aerodynamic diameter of 0.5 μm, the maximum allowable concentrations per cubic meter of air are as follows: ISO Class 1–10 particles, Class 2–100 particles, Class 3–1000 particles, Class 4–10,000 particles, Class 5–100,000 particles, and Class 6–1,000,000 particles. The number of airborne particles is more critical to human health than their total mass, as smaller particles—present in higher quantities—can more easily penetrate the body. Even when the overall mass of particulate matter in the air is low, if it consists of millions of ultrafine particles, the health risk is significantly greater than that posed by fewer, larger particles of equivalent mass [34,35].

According to the literature, hospital indoor air often contains a wide variety of airborne microbes [[36], [37], [38]]. Gram-positive bacteria are generally more abundant than Gram-negative bacteria in these settings [[39], [40], [41]]. This can be attributed to the thicker cell walls of Gram-positive bacteria, which confer greater resistance to environmental stresses [42,43]. To our knowledge, this study is the first to report the presence and diversity of *Nocardia species*—a Gram-positive coccobacillus—in hospital indoor air.

The main origin of *Nocardia species* is soil, and due to its specific enzymes, this bacterium is able to decompose the organic substances of soil [44]. This pathogen causes severe and fatal infections in humans, especially in immunocompromised patients [45]. Nocardiosis includes a wide range of diseases, including pulmonary, abscess, disseminated, and cutaneous infections [46]. The literature review shows that some *Nocardia species* are isolated exclusively from clinical samples, but some species belong to environmental samples [26,47]. However, some species have been isolated from both clinical and environmental samples, which in turn indicates the transmission of these species from soil to air and ultimately to humans via aerosols [48,49].

Airborne microbial contamination in hospitals poses significant risks to patients, particularly those in intensive care units (ICUs), immunocompromised patients, and individuals on immunosuppressive therapies [22]. Additionally, evidence indicates that hospital indoor air may harbor antibiotic-resistant microbes [50,51]. This contamination increases the risk of poor clinical outcomes [52,53]. In this study, we identified *Nocardia species* in hospital indoor air, many of which are known to cause human infections. These findings suggest that microbial contamination of indoor air can serve as a potential source of infection for hospitalized patients [54,55]. The increase in the prevalence of nocardiosis in recent decades indicates a wide awareness of the clinical relevance of *Nocardia species* [56]. The literature indicates that over 40 species of *Nocardia* are involved in human infections, including *N. asteroides* complex, *N. brasiliensis*, *N. abscessus*, *N. cyriacigeorgica*, *N. farcinica*, *N. nova*, *N. otitidiscaviarum*, *N. transvalensis* complex, *N. pseudobrasiliensis*, *N. veterana*, *N. paucivorans*, *N. elegans*, *N. wallacei*, and *N. blacklockiae* [57].

We isolated various *Nocardia species*, including *N. cyriacigeorgica*, *N. asteroides*, *N. otitidiscaviarum*, *N. wallacei*, *N. kroppenstedtii*, *N. farcinica*, and *N. nova*, from different hospital wards: Emergency (*N. cyriacigeorgica*, *N. asteroides*, *N. wallacei*, *N. otitidiscaviarum*), Infectious Diseases (*N. cyriacigeorgica*, *N. asteroides*, *N. farcinica*, *N. nova*), Surgery (*N. cyriacigeorgica*, *N. asteroides*, *N. kroppenstedtii*), Hemodialysis (*N. cyriacigeorgica*, *N. asteroides*), and Orthopedics (*N. cyriacigeorgica*). The emergency ward exhibited the highest species diversity, likely due to high patient turnover, increased staff movement, and frequent air exchange, which can enhance the resuspension and dispersion of dust-borne microorganisms.

Previous studies have reported all isolated species except *N. kroppenstedtii* from patient samples [58,59]. Consistent with earlier findings, *N. cyriacigeorgica* and *N. asteroides* were the most frequently detected species in both clinical and environmental sources [60,61]. These two species are known for their wide environmental distribution, strong desiccation resistance, and association with pulmonary nocardiosis, particularly in immunocompromised hosts [56]. Their predominance in airborne hospital samples may reflect both their ecological adaptability and potential clinical significance. In interpreting our findings, it is important to acknowledge that airborne particulate matter does not necessarily correlate directly with microbial burden. The presence of non-biological particles e.g., dust generated by construction activities, cleaning procedures, or orthopedic practices including plaster cutting may increase measured particulate concentrations without contributing viable microorganisms to the air [62]. Therefore, while bioaerosol sampling detects airborne *Nocardia* and other microbes, particulate matter levels alone cannot be assumed to reflect microbial load or diversity [63]. Our results emphasize the need to distinguish mechanical particle sources from biologically relevant aerosols and support the importance of targeted microbial sampling in healthcare environments rather than relying solely on particulate indicators.

The combination of gravimetric aerosol analysis and culture-based isolation methods was effective in discovering several *Nocardia species*, albeit culture-dependent approaches may underestimate total diversity due to sluggish growth and competition from faster-growing bacteria. Nonetheless, the recovery of seven unique *Nocardia species* indicates the efficacy of our sampling and isolation techniques. The identification of *N. kroppenstedtii* in hospital air is extremely unusual, as this species has rarely been documented outside of environmental or animal sources. Its existence shows that the environment may persist or adapt to indoor microclimates in healthcare facilities.

These results show that bioaerosols containing *Nocardia* can be found in several hospital wards, suggesting possible reservoirs that could lead to opportunistic infections. In high-risk hospital settings, including emergency and infectious disease wards, the preponderance of *N. cyriacigeorgica* and *N. asteroids* species with proven pathogenic potential indicates a need for better air quality control and focused surveillance. While airborne microorganisms represent an important pathway for hospital-acquired infections, it is important to clarify that *Nocardia* constitutes only a small fraction of this overall burden. Most healthcare-associated infections are attributed to more common pathogens such as *Staphylococcus aureus*, Gram-negative bacilli, *Pseudomonas*, or viral respiratory agents, and the contribution of *Nocardia* is comparatively rare and typically limited to immunocompromised patients [56,64]. Thus, our emphasis on *Nocardia* does not imply that it is a major driver of hospital-acquired infection rates; rather, it serves as a useful indicator organism for understanding bioaerosol presence, environmental dissemination, and the potential for opportunistic pathogens to circulate in indoor air [65]. Clarifying this distinction highlights that while improvements in airborne hygiene are urgently needed across healthcare settings, the risk posed by *Nocardia* specifically represents a focused but important component of a much broader infection-control challenge.

Several contextual factors may explain the high diversity of *Nocardia species* detected in this study. Iranshahr is located in a tropical region characterized by warm temperatures, seasonal humidity, and frequent dust storms, all of which can facilitate the aerosolization of soil-derived *Nocardia* strains and increase their airborne persistence [56,66]. In addition, inadequate sanitation practices, accumulation of dust, and insufficient air filtration systems in some hospital areas can elevate the microbial load in indoor air [67,68]. Limited filtration control and aging infrastructure may further enable the circulation and survival of airborne pathogens within enclosed hospital environments [69]. Together, these factors create favorable conditions for the transmission of *Nocardia* through bioaerosols and highlight the importance of environmental monitoring and infection-control interventions in healthcare settings of developing regions. Although understanding airborne microbial profiles may contribute to infection prevention strategies, it is important to acknowledge that this concept applies unevenly across pathogens. In particular, *Nocardia* grows slowly and typically requires prolonged culture incubation and specialized laboratory methods; therefore, early clinical detection based solely on culture is unlikely [70]. Our argument is not that airborne monitoring will enable rapid diagnosis of nocardiosis in individual patients, but rather that identifying *Nocardia* in the hospital air environment may serve as an epidemiological signal of potential exposure risk, especially for immunocompromised populations. In this sense, airborne surveillance functions as a preventive environmental assessment tool rather than a rapid diagnostic strategy, complementing rather than replacing clinical detection methods [71,72].

To the best of our knowledge, this is the first study to report the detection and isolation of clinically relevant *Nocardia species* from hospital indoor air and supplies, particularly within a healthcare setting in a developing country. Previous research on *Nocardia* has primarily focused on clinical specimens, with minimal investigation into hospital environments or airborne sources. The present findings, therefore, provide novel evidence supporting the role of environmental reservoirs as a potential source of *Nocardia* exposure in healthcare facilities. This study presents one of the earliest documented reports on the presence of clinically significant *Nocardia species* in hospital indoor air. *Nocardia species* are known to cause a wide range of infections and exhibit varying drug susceptibility patterns [73]. Accurate species-level identification is essential for optimal therapeutic management [56]. However, the slow-growing nature of *Nocardia* often complicates its isolation and identification. Traditional laboratory methods, such as partial acid-fast staining and enzymatic hydrolysis assays, are labor-intensive and may yield inconclusive results [74]. Consequently, the clinical relevance of *Nocardia species* has often been overlooked. Our findings complement recent metagenomic investigations showing that hospital air can contain diverse microbiomes and ARGs, and together these data highlight the importance of routine environmental air monitoring particularly in low-resource settings where occult environmental reservoirs may pose unrecognized risks to vulnerable patients [75,76].

In this study, molecular techniques were employed for species-level identification, revealing a diversity of *Nocardia species* in hospital environments similar to those found in clinical samples in Iran. This study had several limitations: It was conducted over a short period, without accounting for seasonal variations. The airborne microbial load was not quantified. The sample size was small, and only one hospital was analyzed. Future long-term studies are needed to strengthen the findings and further explore the clinical relevance of airborne microbial contamination.

## 5 Conclusion

The assessment of airborne bacteria in hospital indoor air is crucial, although standardized guidelines for acceptable microbial loads are lacking. The presence of pathogenic microbes in hospital indoor air underscores the need for effective filtration and air filtration systems, as well as comprehensive disinfection strategies. Contaminated hospital environments can act as sources of opportunistic infections for vulnerable patients. This study supports the implementation of continuous microbial surveillance and decontamination measures in hospital settings as an effective strategy for infection control and prevention.

In order to improve indoor air quality and successfully remove particulate matter and bioaerosols, hospital ventilation systems are advised to use HEPA and ULPA filters. The use of positive and negative air pressure systems, positive pressure in hygienic settings like operating rooms, and negative pressure in contaminated areas like hospital pathology labs is another crucial component in enhancing hospital air quality. These findings underscore the necessity for additional research to establish clinical correlations and assess the effectiveness of routine bioaerosol monitoring in infection prevention programs.

## CRediT authorship contribution statement

**Zahed Ahmadi:** Writing – review & editing, Writing – original draft, Supervision, Methodology, Investigation, Formal analysis, Data curation, Conceptualization. **Alireza Moradabadi:** Writing – review & editing. **Sara Kamal-Shasavar:** Writing – review & editing, Writing – original draft, Software, Methodology, Investigation, Formal analysis, Data curation.

## Consent to participate (missing)

‘This is not applicable.

## Consent to publish (missing)

This is not applicable.

## Ethical approval

This study was approved by the Research Ethics Committee of Iranshahr University of Medical Sciences (Ethical code: IR. IRSHUMS.REC.1400.017). The authors certify that all data collected during the study are as presented in this paper, and no data from the study has been or will be published elsewhere separately.

## Data sharing

Data are available at the correspond requests.

## Declaration of competing interest

The authors declare no financial or personal conflicts of interest related to this study on microbial contamination in hospital environments.

## References

[1] Fernandes J.J.D. (2022).

[2] Judson S.D., Munster V.J. (2019). Nosocomial transmission of emerging viruses via aerosol-generating medical procedures. Viruses.

[3] Stockwell R.E. (2019). Indoor hospital air and the impact of ventilation on bioaerosols: a systematic review. J Hosp Infect.

[4] Faghihi-Zarandia A. (2022). Determination of mercury values in urine and air of chloralkali workers by copper nanoparticles functionalized in carboxylic carbon nanotubes and the effects of mercury exposure on oxidative stress. Analytical Methods in Environmental Chemistry Journal.

[5] Rabbani Y. (2025). Air quality and hospital-acquired infections: a case Study of ventilation and bioaerosols in an educational Hospital. Health Scope.

[6] Koehler K.A., Peters T.M. (2015). New methods for personal exposure monitoring for airborne particles. Curr Environ Health Rep.

[7] Pan M., Lednicky J.A., Wu C.Y. (2019). Collection, particle sizing and detection of airborne viruses. J Appl Microbiol.

[8] Chen L. (2024). Pathogenic bacteria and fungi in bioaerosols from specialized hospitals in Shandong province, East China. Environ Pollut.

[9] Saubolle M.A., Sussland D. (2003). Nocardiosis: review of clinical and laboratory experience. J Clin Microbiol.

[10] Brown-Elliott B.A. (2006). Clinical and laboratory features of the Nocardia spp. based on current molecular taxonomy. Clin Microbiol Rev.

[11] Fujita T. (2016). Clinical characteristics of pulmonary nocardiosis in immunocompetent patients. J Infect Chemother.

[12] Kontogiorgi M. (2013). Pulmonary nocardiosis in an immunocompetent patient with COPD: the role of defective innate response. Heart Lung.

[13] Kageyama A. (2004). Nocardial infections in Japan from 1992 to 2001, including the first report of infection by Nocardia transvalensis. Eur J Epidemiol.

[14] Mahendra P., Dave P. (2016). Nocardiosis: an emerging infectious actinomycetic disease of humans and animals. J Microbiol Microb Technol.

[15] Nzeusseu Toukap A. (1996). Nocardiosis: a rare cause of pleuropulmonary disease in the immunocompromised host. Acta Clin Belg.

[16] Motallebirad T. (2024). Fifteen years of phenotypic and genotypic surveillance and antibiotic susceptibility pattern of actinomycetes (Mycobacterium, Nocardia, Rhodococcus, etc.) in clinical and environmental samples of Iran. Diagn Microbiol Infect Dis.

[17] O'Brien A. (2024). Nocardia species distribution and antimicrobial susceptibility within Australia. Intern Med J.

[18] Kageyama A. (2004). Nocardial infections in Japan from 1992 to 2001, including the first report of infection by Nocardia transvalensis. Eur J Epidemiol.

[19] Han Y. (2024). Clinical characteristics and drug resistance of Nocardia in Henan, China, 2017–2023. Ann Clin Microbiol Antimicrob.

[20] Boyce J.M. (2008). Impact of hydrogen peroxide vapor room decontamination on Clostridium difficile environmental contamination and transmission in a healthcare setting. Infect Control Hosp Epidemiol.

[21] Beggs C. (2015). Environmental contamination and hospital‐acquired infection: factors that are easily overlooked. Indoor Air.

[22] Belay M.M. (2024). Investigating microbial contamination of indoor air, environmental surfaces, and medical equipment in a Southwestern Ethiopia Hospital. Environ Health Insights.

[23] Sikora A., Zahra F. (2020).

[24] Wu D. (2022). Inhalable antibiotic resistomes emitted from hospitals: metagenomic insights into bacterial hosts, clinical relevance, and environmental risks. Microbiome.

[25] Klvanova E. (2024). Resistome in the indoor dust samples from workplaces and households: a pilot study. Front Cell Infect Microbiol.

[26] Duggal S.D., Chugh T.D. (2020). Nocardiosis: a neglected disease. Med Princ Pract.

[27] Khaledi C.K.A. (2017). A Case study of evaluation and distribution of tourism climate by using TCI: baluchestan region of Iran. Open J Geol.

[28] Bafghi M.F. (2014). DNA extraction from nocardia species for special genes analysis using PCR. N Am J Med Sci.

[29] Madueño L. (2011). Isolation and characterization of indigenous soil bacteria for bioaugmentation of PAH contaminated soil of semiarid Patagonia, Argentina. Int Biodeterior Biodegrad.

[30] Stackebrandt E., Rainey F.A., Ward-Rainey N.L. (1997). Proposal for a new hierarchic classification system, Actinobacteria classis Nov. Int J Syst Evol Microbiol.

[31] Verde S.C. (2015). Microbiological assessment of indoor air quality at different hospital sites. Res Microbiol.

[32] Baurès E. (2018). Indoor air quality in two French hospitals: measurement of chemical and microbiological contaminants. Sci Total Environ.

[33] Nevalainen A., Morawaska L. (2003). Biological agents in indoor environments. Assessment of health risks. Work conducted by a WHO Expert Group between,.

[34] Kwon H.-S., Ryu M.H., Carlsten C. (2020). Ultrafine particles: unique physicochemical properties relevant to health and disease. Exp Mol Med.

[35] Whyte W. (2024). Classification of air cleanliness by particle concentration according to ISO 14644-1. Clean Air and Containment Review.

[36] Blomquist G. (1994). Sampling of biological particles. Analyst.

[37] Gizaw Z., Gebrehiwot M., Yenew C. (2016). High bacterial load of indoor air in hospital wards: the case of University of Gondar teaching hospital, Northwest Ethiopia. Multidisciplinary respiratory medicine.

[38] Fekadu S., Getachewu B. (2015). Microbiological assessment of indoor air of teaching hospital wards: a case of Jimma University specialized hospital. Ethiopian journal of health sciences.

[39] Sudharsanam S. (2009). Microorganisms in bioaerosols in indoor air of hospital and non-hospital settings. Sri Ramachandra Journal of Medicine.

[40] Qudiesat K. (2009). Assessment of airborne pathogens in healthcare settings. Afr J Microbiol Res.

[41] Kotgire S. (2020). Bioaerosol assessment of indoor air in hospital wards from a tertiary care hospital. Indian J Microbiol Res.

[42] Rohde M. (2019). The Gram-positive bacterial cell wall. Microbiol Spectr.

[43] Beveridge T.J. (1999). Structures of gram-negative cell walls and their derived membrane vesicles. J Bacteriol.

[44] Li S. (2017). Nocardia tengchongensis sp. Nov., isolated from a soil sample. Antonie Leeuwenhoek.

[45] Kandi V. (2015). Human Nocardia infections: a review of pulmonary nocardiosis. Cureus.

[46] Mishra S., Randhawa H. (1969). Application of paraffin bait technique to the isolation of Nocardia asteroides from clinical specimens. Appl Microbiol.

[47] Fatahi-Bafghi M. (2018). Nocardiosis from 1888 to 2017. Microb Pathog.

[48] Ambrosioni J., Lew D., Garbino J. (2010). Nocardiosis: updated clinical review and experience at a tertiary center. Infection.

[49] Wang H.-L. (2014). Nocardiosis in 132 patients with cancer: microbiological and clinical analyses. Am J Clin Pathol.

[50] Baudet A. (2021). Indoor air quality in healthcare and care facilities: chemical pollutants and microbiological contaminants. Atmosphere.

[51] Lee G., Yoo K. (2022). A review of the emergence of antibiotic resistance in bioaerosols and its monitoring methods. Rev Environ Sci Biotechnol.

[52] Kayta G. (2022). Indoor air microbial load, antibiotic susceptibility profiles of bacteria, and associated factors in different wards of Arba Minch General Hospital, southern Ethiopia. PLoS One.

[53] Douglas S.I., Robinson V. (2019). Indoor microbiological air quality in some wards of a tertiary health institution in Port Harcourt, Nigeria. Journal of Pharmacy and Biological Sciences.

[54] Wang H. (2022). Epidemiology and antimicrobial resistance profiles of the Nocardia species in China, 2009 to 2021. Microbiol Spectr.

[55] Jayashankar C. (2024). Neglected tropical diseases: a comprehensive review. Cureus.

[56] Traxler R.M. (2022). Updated review on Nocardia species: 2006–2021. Clin Microbiol Rev.

[57] Lebeaux D. (2019). Antibiotic susceptibility testing and species identification of Nocardia isolates: a retrospective analysis of data from a French expert laboratory, 2010–2015. Clin Microbiol Infection.

[58] Hashemi-Shahraki A. (2015). Genetic diversity and antimicrobial susceptibility of Nocardia species among patients with nocardiosis. Sci Rep.

[59] Fatahi-Bafghi M. (2016). Comparison of restriction enzyme pattern analysis and full gene sequencing of 16S rRNA gene for Nocardia species identification, the first report of Nocardia transvalensis isolated of sputum from Iran, and review of the literature. Antonie Leeuwenhoek.

[60] Javadi A. (2019). Diversity of Nocardia species isolated from transplantation and cancer centers of Tehran hospitals. Journal of Acute Disease.

[61] Motalebirad T., Rahdar H.A. (2019). Isolation and molecular characterization of hydrocarbon degrading nocardia isolated from Hospital environments in Isfahan Province. Infection Epidemiology and Microbiology.

[62] AlRayess S. (2022). Airborne bacterial and PM characterization in intensive care units: correlations with physical control parameters. Air Qual Atmos Health.

[63] Seo J.H. (2020). Prediction model for airborne microorganisms using particle number concentration as surrogate markers in hospital environment. Int J Environ Res Publ Health.

[64] Haque M. (2018). Health care-associated infections–an overview. Infect Drug Resist.

[65] Saleem Z. (2019). Point prevalence surveys of health-care-associated infections: a systematic review. Pathog Glob Health.

[66] Najafi M.S., Alizadeh O. (2023). Climate zones in Iran. Meteorol Appl.

[67] Berihun G. (2025). Prevalence of respiratory symptoms and associated factors among sanitation workers in Sub Saharan Africa: a systematic review and meta-analysis. Front Public Health.

[68] Ashuro Z. (2022). Assessment of microbiological quality of indoor air at different hospital sites of Dilla University: a cross-sectional study. Environ Health Insights.

[69] Beggs C.B. (2024). Airborne transmission of SARS-CoV-2: the contrast between indoors and outdoors. Fluid.

[70] Conville P.S. (2018). The complexities of Nocardia taxonomy and identification. J Clin Microbiol.

[71] Wauters G. (2005). Distribution of Nocardia species in clinical samples and their routine rapid identification in the laboratory. J Clin Microbiol.

[72] Huang L. (2019). Clinical features, identification, antimicrobial resistance patterns of Nocardia species in China: 2009–2017. Diagn Microbiol Infect Dis.

[73] Margalit I. (2021). How do I manage nocardiosis?. Clin Microbiol Infection.

[74] Conville P.S., Witebsky F.G. (2005). Multiple copies of the 16S rRNA gene in Nocardia nova isolates and implications for sequence-based identification procedures. J Clin Microbiol.

[75] Habibi N. (2022). Bacterial and fungal communities in indoor aerosols from two Kuwaiti hospitals. Front Microbiol.

[76] Amato P. (2023). The aeromicrobiome: the selective and dynamic outer-layer of the Earth's microbiome. Front Microbiol.

